# Association between adverse pregnancy outcomes and incidence of advanced cardiovascular-kidney-metabolic disease: a population-based cohort study

**DOI:** 10.3389/fcvm.2026.1859791

**Published:** 2026-08-21

**Authors:** Yifan Wang, Changhao Zu, Dantong Wang, Nandi Liu, Meichen Yang, Yufeng Bian, Wenli Yin, Yuntao Wu, Chunyu Ruan, Kai Cui, Lihua Chen, Rui Geng, Haiyan Zhao

**Affiliations:** 1 Kailuan General Hospital Affiliated with North China University of Science and Technology, Tangshan, China; 2 North China University of Science and Technology, Tangshan, China; 3 Department of Cardiology, Kailuan General Hospital, Tangshan, China; 4 Hebei North University, Zhangjiakou, China; 5 Kailuan General Hospital, Tangshan, China

**Keywords:** adverse pregnancy outcomes, cardiovascular, cardiovascular-kidney-metabolic, cohort study, correlational study

## Abstract

**Objective:**

Adverse pregnancy outcomes (APOs) are independent risk factors for cardiovascular disease in women. Within the novel cardiovascular-kidney-metabolic (CKM) framework, evidence linking APOs to advanced CKM (stages 3–4) remains limited.

**Methods:**

This study used data from the Kailuan Cohort and combined cross-sectional and longitudinal designs. The cross-sectional analysis included 2,738 women with singleton deliveries. For the longitudinal analysis, 2,575 women without advanced CKM at baseline were followed up for a median of 11.68 years. A history of APOs was the primary exposure. Multinomial logistic regression and Cox proportional hazards models were used.

**Results:**

Cross-sectionally, APOs were positively associated with baseline CKM stage severity, with odds ratios increasing across stages (Stage 1: odds ratio [OR] 1.42, 95% confidence interval [CI] 1.03–1.96; Stage 2: OR 1.65, 95% CI 1.26–2.17; advanced CKM: OR 2.93, 95% CI 1.74–4.94). Women with APOs had a higher incidence of advanced CKM than those without (13.39 vs. 8.42 per 1,000 person-years). After multivariable adjustment, a history of APOs was associated with a 27% increased risk of incident advanced CKM (hazard ratio 1.27, 95% CI 1.01–1.61). Sensitivity analyses excluding participants with less than 4 years of follow-up and those taking medications at baseline yielded results consistent with the primary analysis. In the exploratory analyses, we further evaluated the association between a history of APOs and incident CKM stages 3 and 4. A history of APOs was associated with an increased risk of incident CKM Stage 4, whereas no statistically significant association was observed with incident CKM Stage 3.

**Conclusion:**

A history of APOs was associated with greater baseline CKM severity and an increased risk of developing advanced CKM. Therefore, APOs may serve as early markers for identifying women at risk of progressive CKM.

## Introduction

Pregnancy is a critical physiological period with profound and lasting implications for cardiometabolic health (1). Recognized as a distinct sex-specific risk factor, it induces hemodynamic, metabolic, and renal adaptations that collectively serve as a natural “stress test” for the cardiovascular-kidney-metabolic (CKM) syndrome (2). When the maternal adaptive capacity is exceeded, this may manifest as adverse pregnancy outcomes (APOs), including hypertensive disorders of pregnancy (HDP), gestational diabetes mellitus (GDM), preterm birth (PTB), and low birth weight (LBW), which are increasingly viewed as early clinical markers of underlying multisystem vulnerability (3, 4).

Robust evidence has confirmed that APOs are associated with an increased risk of developing single-system diseases later in life. Specifically, women with prior HDP have a 1.28- to 1.93-fold increased likelihood of developing cardiovascular disease (CVD), chronic kidney disease (CKD), and type 2 diabetes mellitus (T2DM) compared to those with normotensive pregnancies (5–7). Similarly, a history of GDM is associated with a 1.72–7.1-fold increase in the risk of these conditions (8–10). Furthermore, PTB and LBW have been consistently associated with long-term maternal cardiometabolic disturbances including hypertension and insulin resistance (11, 12). Collectively, these observations underscore the fact that APOs are early clinical indicators of sustained vulnerability to cardiovascular, renal, and metabolic disorders in women. Potential mechanisms include endothelial dysfunction, inflammatory dysregulation, insulin resistance, and renal microvascular injury. Alterations in DNA methylation, histone modifications, and non-coding RNA regulation may further contribute to long-term CKM susceptibility following pregnancy complications (3, 13).

Cardiometabolic organ damage seldom occurs in isolation. Rather, it evolves as a progressive, multisystem process characterized by reciprocal interactions and a shared pathophysiology. To capture this clinical continuum, the American Heart Association (AHA) recently advanced the integrated concept of CKM, emphasizing coordinated risk assessment and management across the three systems (14). Epidemiological data indicate a high prevalence of CKM among Chinese adults, with recent estimates increasing from 77.1% to 83.7% (15). Although sex differences in CKM prevalence are well-established, growing evidence suggests that women with CKM experience higher CKM-related mortality rates (16). While research on CKM in women has predominantly focused on traditional risk factors, such as hormone profiles and body fat distribution (17, 18), female-specific early risk indicators, particularly APOs, remain poorly understood. Consequently, evidence clarifying the association between a history of APOs and progression of multisystem CKM remains sparse, thereby limiting the development of targeted sex-stratified prevention strategies.

To address this critical gap in evidence, we conducted a population-based study using both cross-sectional and longitudinal data from the Kailuan Cohort. This study aimed to examine the association between APOs and the severity of CKM staging, as well as the risk of advanced CKM. Our findings may inform the development of sex-specific, pregnancy-history-aware strategies for the early identification and prevention of CKM in women.

## Methods

### Study population

**Figure 1:**
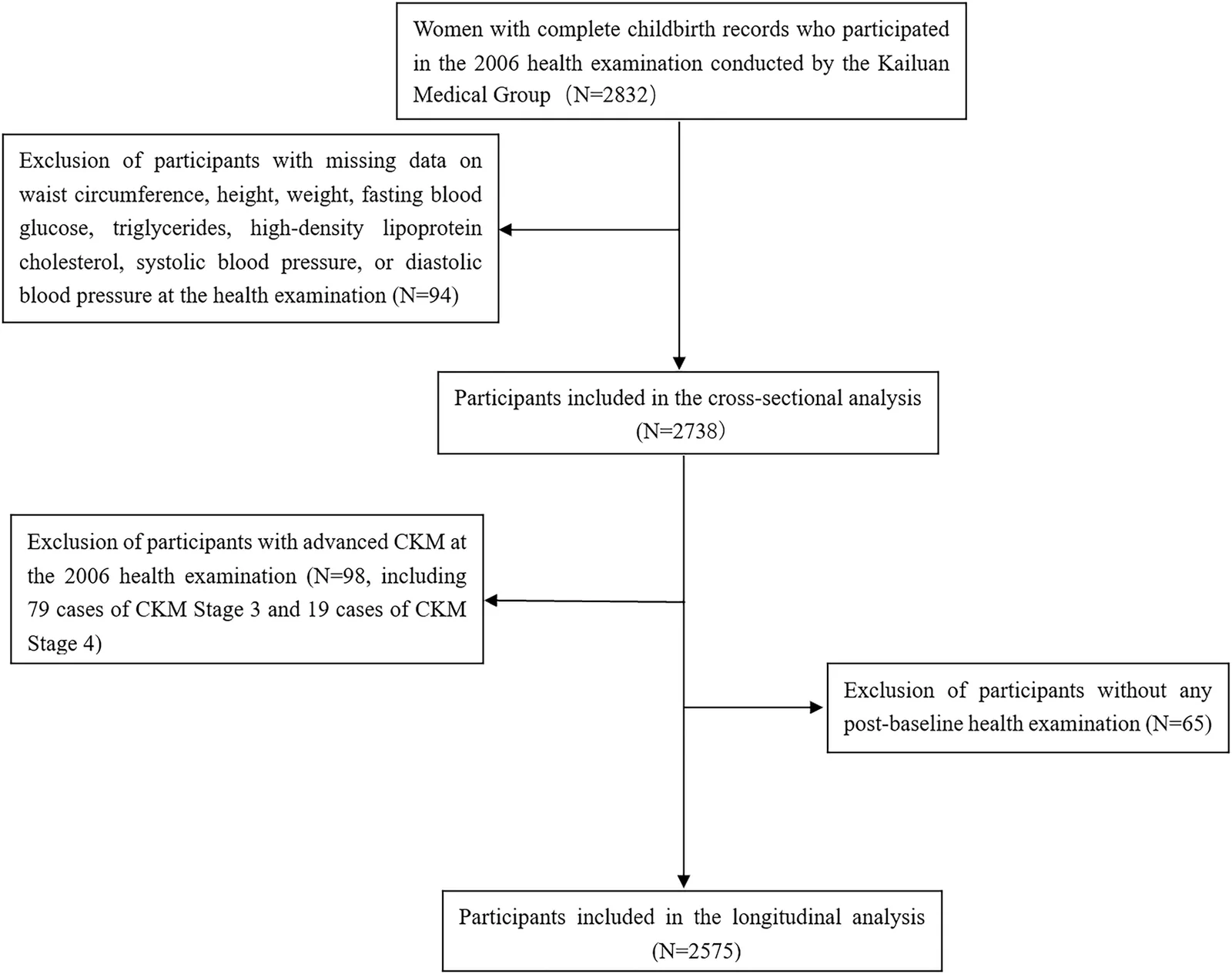
Flowchart for the study population.

Data were obtained from the Kailuan Study (Clinical Trial Registration No.: ChiCTR-TNRC-11001489). Participants were women who completed the 2006 health examination conducted by the Kailuan Medical Group and had complete childbirth records. The cross-sectional analysis included 2,738 participants with complete data on CKM diagnoses at the 2006 examination. For the longitudinal analysis, we first excluded participants with advanced CKM at baseline and subsequently excluded those without any post-baseline health examination, resulting in a final cohort of 2,575 participants **(**Figure 1**)**. The study was conducted in accordance with the Declaration of Helsinki and was approved by the Ethics Committee of Kailuan General Hospital (No. 2024017). All the participants provided written informed consent.

### Data collection

Obstetric data were extracted from hospitalization records, including history of hypertension and diabetes, age at delivery, and maternal and neonatal complications.

Sociodemographic and lifestyle information included age, education level, income level, salt intake, sleep duration, and physical activity level. The income level was dichotomized as < 800 CNY per month or ≥ 800 CNY per month. Salt intake was derived from responses to the questionnaire on salt preference, with response options of “light (< 6 g/d)”, “fair (6–10 g/d)”, or “salty (> 10 g/d)”. Physical activity was defined as exercising at least three times per week for at least 30 min each time.

Data on medical conditions, including hypertension, diabetes, and dyslipidemia, as well as medication use were obtained from self-reported questionnaires, medical records, and physical examinations.

Height, weight, waist circumference (WC), and blood pressure were measured by trained physicians according to standardized protocols.

Fasting blood glucose (FBG), low-density lipoprotein cholesterol (LDL-C), high-density lipoprotein cholesterol (HDL-C), triglycerides (TG), total cholesterol (TC), high-sensitivity C-reactive protein (hs-CRP), uric acid (UA), and serum creatinine (Scr) were measured using an automatic analyzer (Hitachi 747, Tokyo, Japan). The estimated glomerular filtration rate (eGFR) was calculated using the Chronic Kidney Disease Epidemiology Collaboration equation (19). A single random midstream morning urine sample was collected from each participant during the interview, and all urine samples were analyzed using a urine analyzer (N-600; Changchun Dirui Medical Technology Co., Ltd.) at the central laboratory of Kailuan General Hospital. Semi-quantitative proteinuria results were recorded as negative (< 15 mg/dL), trace (15–29 mg/dL), 1+ (30–300 mg/dL), 2+ (300–1,000 mg/dL), or 3+ (> 1,000 mg/dL).

### Definition of adverse pregnancy outcomes

APOs were defined as the presence of any one or more of the following conditions: (1) HDP (20): 1) gestational hypertension, defined as hypertension occurring for the first time after 20 weeks of pregnancy, with systolic blood pressure (SBP) ≥ 140 mmHg and/or diastolic blood pressure (DBP) ≥ 90 mmHg and a negative proteinuria test; 2) preeclampsia-eclampsia, defined as SBP ≥ 140 mmHg and/or DBP ≥ 90 mmHg after 20 weeks of pregnancy accompanied by a positive urine protein test; (2) GDM (21): FBG ≥ 5.1 mmol/L occurring for the first time during pregnancy; (3) PTB (22): neonate with a gestational age less than 37 weeks at birth; (4) LBW (22): neonate with a birth weight < 2,500 g.

### Cardiovascular-kidney-metabolic risk factors

Hypertension was defined as a self-reported history of hypertension, current antihypertensive treatment, SBP ≥ 140 mmHg, and/or DBP ≥ 90 mmHg. Diabetes was defined as a self-reported history of diabetes, current hypoglycemic treatment, or FBG ≥ 7.0 mmol/L. CKD was classified into four CKD risk levels: low, moderately increased, high, and very high, according to the Kidney Disease Improving Global Outcomes 2012 recommendations (23). CVD was defined as heart failure; atrial fibrillation; myocardial infarction; or stroke, including cerebral infarction, intracerebral hemorrhage, and subarachnoid hemorrhage. All aforementioned events were confirmed by specialist physicians based on inpatient medical records.

Metabolic syndrome was defined as the presence of three or more of the following conditions (24): (1) WC ≥ 80 cm in women; (2) HDL-C ≤ 1.3 mmol/L in women; (3) TG ≥ 1.7 mmol/L or use of lipid-lowering medications; (4) elevated blood pressure (SBP ≥ 130 mmHg and/or DBP ≥ 80 mmHg, use of antihypertensive medications, or a prior diagnosis of hypertension); and (5) FBG ≥ 5.6 mmol/L, use of hypoglycemic medications, or a prior diagnosis of diabetes.

### Definition of cardiovascular-kidney-metabolic stages

According to a scientific statement from the AHA, CKM is classified into distinct stages, ranging from Stage 0 to Stage 4 (25): Stage 0 was characterized by the absence of CKM risk factors, including normal body mass index (BMI) and WC, normoglycemia, normotension, a normal lipid profile, and no signs of CKD or subclinical or clinical CVD. Stage 1 was defined as having excess weight (BMI ≥ 23 kg/m^2^), abdominal obesity (WC ≥ 80 cm in women), or dysfunctional adipose tissue, clinically manifested as impaired glucose tolerance or prediabetes (5.6 mmol/L ≤ FBG ≤ 6.9 mmol/L), in the absence of additional metabolic risk factors or CKD. Stage 2 was defined as the presence of metabolic risk factors (hypertriglyceridemia ≥ 1.5 mmol/L, hypertension, metabolic syndrome, diabetes), moderate- or high-risk CKD, or both. Stage 3 was defined as subclinical CVD. Stage 3 CKM included individuals with a high predicted CVD risk or very high-risk CKD calculated using the prediction for atherosclerotic Cardiovascular Disease risk in China (China-PAR) model, which is specific to China (26). Risk equivalents were used to define this stage due to the absence of related indicators. Stage 4 was defined as clinical CVD in individuals with excess or dysfunctional adiposity, other metabolic risk factors, or CKD. Advanced CKM was defined as Stage 3 or 4.

### Ascertainment of outcomes

Advanced CKM was defined as the endpoint. The baseline was defined as the 2006 examination. Follow-up continued until the first occurrence of advanced CKM or until the participant's last available health examination before the administrative censoring date of December 31, 2023, whichever came first.

### Statistical analysis

Participants were stratified according to the occurrence of APOs. Continuous variables were summarized as means (standard deviations) or medians (interquartile ranges [IQRs]) as appropriate. Categorical variables were presented as frequencies and percentages. The distributions of characteristics were compared between groups using independent t-tests for normally distributed continuous variables, Mann–Whitney *U* tests for non-normally distributed continuous variables, and *χ*^2^ tests for categorical variables. Missing covariate data were imputed using multiple imputation with chained equations.

For the cross-sectional analysis, CKM stage was modeled as a four-category dependent variable (Stage 0, Stage 1, Stage 2, and advanced CKM), with Stage 0 as the reference category, and APO history as the primary independent variable. The proportional odds assumption was not satisfied in the parallel-lines test; therefore, multinomial logistic regression was used to estimate odds ratios (ORs) and 95% confidence intervals (CIs). Three sequential models were used in this study. Model 1 was unadjusted. Model 2 was adjusted for age at delivery. Model 3 was further adjusted for age at baseline, LDL-C, hs-CRP, UA, income level, salt intake, physical activity, and sleep duration. No significant multicollinearity was detected in any model.

In the longitudinal analysis, we calculated the incidence of advanced CKM (per 1,000 person-years) and used Cox proportional hazards regression to evaluate the association between a history of APOs and incident advanced CKM, reporting hazard ratios (HRs) with 95% CIs. The same covariates as those used in the cross-sectional analysis were adjusted for. Cumulative incidence was visualized using Kaplan–Meier curves and compared using the log-rank test. To assess the robustness of the findings, we conducted two sensitivity analyses: first, excluding participants with less than 4 years of follow-up and second, excluding participants using antihypertensive, glucose-lowering, or lipid-lowering medications at baseline. In addition, incident CKM stages 3 and 4 were evaluated separately in exploratory stage-specific outcome analyses. Exploratory subgroup analyses were conducted by baseline age (< 45 years or ≥ 45 years), BMI (< 24 kg/m^2^ or ≥ 24 kg/m^2^), education level (below high school or high school or above), and income level (< 800 CNY/month or ≥ 800 CNY/month). Interaction terms between a history of APOs and each stratification variable were included to formally test for effect modification, with the corresponding *p* values reported.

All statistical analyses were performed using SAS 9.4 (SAS Institute Inc., Cary, NC, USA). Statistical significance was set at a two-sided *p* value < 0.05.

## Results

### Participant characteristics

A total of 2,738 women were included in this cross-sectional analysis. The mean age at delivery was 27.4 ± 4.3 years, and the mean age at baseline was 40.8 ± 7.8 years. Compared to the non-APOs group, the APOs group showed no significant difference in age at delivery but had a higher mean age at baseline, as well as higher levels of WC, SBP, DBP, TC, TG, FBG, and BMI. The proportions of participants with lower income and lower education were significantly higher in the APOs group, along with a higher prevalence of hypertension **(**Table 1**).** Baseline characteristics according to CKM stage are presented in Supplementary Table S1**.**

**Table 1:** Baseline characteristics of the study population according to different groups.

Characteristics	Total (*N* = 2,738)	APOs (*N* = 916)	Non-APOs (*N* = 1,822)	*p*-value
Delivery age, years	27.4 ± 4.3	27.5 ± 4.3	27.4 ± 4.3	0.659
Baseline age, years	40.8 ± 7.8	42.4 ± 7.8	40.1 ± 7.6	<0.001
WC, cm	80.9 ± 10.8	82.7 ± 10.8	80.0 ± 10.7	<0.001
SBP, mmHg	117.4 ± 18.3	121.1 ± 19.6	115.5 ± 17.4	<0.001
DBP, mmHg	77.0 ± 10.9	79.0 ± 11.6	76.0 ± 10.4	<0.001
HDL-C, mmol/L	1.5 ± 0.3	1.5 ± 0.4	1.5 ± 0.3	0.348
LDL-C, mmol/L	2.1 ± 0.7	2.1 ± 0.7	2.1 ± 0.7	0.342
TC, mmol/L	4.7 ± 0.9	4.8 ± 1.0	4.7 ± 0.9	0.008
TG, mmol/L	1.0 (0.7, 1.4)	1.0 (0.7, 1.5)	1.0 (0.7, 1.4)	0.025
UA, μmol/L	232.3 ± 57.5	232.2 ± 57.3	232.3 ± 57.6	0.671
FBG, mmol/L	5.2 ± 1.5	5.3 ± 1.7	5.1 ± 1.4	0.003
BMI, kg/m^2^	23.9 ± 3.6	24.5 ± 3.7	23.6 ± 3.6	<0.001
hs-CRP, mg/L	0.5 (0.2, 1.3)	0.5 (0.2, 1.6)	0.5 (0.2, 1.2)	0.073
Income level ≥ 800 CNY/month, *n* (%)	357 (13.0%)	75 (8.2%)	282 (15.5%)	<0.001
Physical activity, *n* (%)	121 (4.4%)	41 (4.5%)	80 (4.4%)	0.919
High school or above, *n* (%)	938 (34.3%)	229 (25.0%)	709 (38.9%)	<0.001
Salt intake, *n* (%)				0.121
< 6 g/d	183 (6.7%)	59 (6.4%)	124 (6.8%)	
6–10 g/d	2,385 (87.1%)	812 (88.6%)	1,573 (86.3%)	
> 10 g/d	170 (6.2%)	45 (4.9%)	125 (6.9%)	
Sleep duration, *n* (%)				0.132
≤ 6 h/d	256 (9.3%)	73 (8.0%)	183 (10.0%)	
6–8 h/d	2,428 (88.7%)	828 (90.4%)	1,600 (87.8%)	
> 8 h/d	54 (2.0%)	15 (1.6%)	39 (2.1%)	
Hypertension, *n* (%)	499 (18.2%)	218 (23.8%)	281 (15.4%)	<0.001
Diabetes, *n* (%)	125 (4.6%)	50 (5.5%)	75 (4.1%)	0.112

Values are mean (standard deviation), median (interquartile range), or *n* (%) as appropriate.

BMI, body mass index; SBP, systolic blood pressure; DBP, diastolic blood pressure; FBG, fasting blood glucose; UA, uric acid; HDL-C, high-density lipoprotein cholesterol; LDL-C, low-density lipoprotein cholesterol; hs-CRP, high-sensitivity C-reactive protein; TC, total cholesterol; TG, triglycerides; WC, waist circumference.

APOs, adverse pregnancy outcomes.

### Cross-sectional association between adverse pregnancy outcomes and cardiovascular-kidney-metabolic stages

In the cross-sectional analysis, the CKM stage distribution differed significantly between the two groups (*P* < 0.001). The prevalence rates of CKM stages 0, 1, and 2, and advanced CKM in the non-APOs group were 18.72%, 15.86%, 62.95%, and 2.47%, respectively, and the corresponding rates in the APOs group were 9.93%, 13.65%, 70.63%, and 5.79%, respectively **(**Table 2**)**. After multivariable adjustment, with CKM Stage 0 as the reference category, a history of APOs was associated with higher odds of CKM Stage 1 (OR, 1.42; 95% CI, 1.03–1.96), CKM Stage 2 (OR, 1.65; 95% CI, 1.26–2.17), and advanced CKM (OR, 2.93; 95% CI, 1.74–4.94) **(**Table 3**).**

**Table 2:** The distribution of CKM stages in different groups.

Group	CKM 0	CKM 1	CKM 2	Advanced CKM	*χ*2	*P*-value
Non-APOs	341 (18.72%)	289 (15.86%)	1,147 (62.95%)	45 (2.47%)	55.98	<0.001
APOs	91 (9.93%)	125 (13.65%)	647 (70.63%)	53 (5.79%)		

APOs, adverse pregnancy outcomes; CKM, cardiovascular-kidney-metabolic.

**Table 3:** Multinomial logistic regression analysis of the association between APOs and different CKM stages.

Model	CKM 0	CKM 1		CKM 2		Advanced CKM	
		OR (95% CI)	P	OR (95% CI)	P	OR (95% CI)	P
APOs							
Model 1	Reference	1.62 (1.19–2.22)	0.002	2.11 (1.65–2.72)	<0.001	4.41 (2.79–6.99)	<0.001
Model 2	Reference	1.63 (1.19–2.23)	0.002	2.13 (1.66–2.74)	<0.001	4.80 (2.98–7.73)	<0.001
Model 3	Reference	1.42 (1.03–1.96)	0.031	1.65 (1.26–2.17)	<0.001	2.93 (1.74–4.94)	<0.001

Model 1 was unadjusted. Model 2 was adjusted for age at delivery. Model 3 was further adjusted for age at baseline, low-density lipoprotein cholesterol, high-sensitivity C-reactive protein, uric acid, income level, salt intake, physical activity, and sleep duration based on Model 2.

APOs, adverse pregnancy outcomes; CKM, cardiovascular-kidney-metabolic.

### Longitudinal association between adverse pregnancy outcomes and incident advanced cardiovascular-kidney-metabolic disease

**Figure 2:**
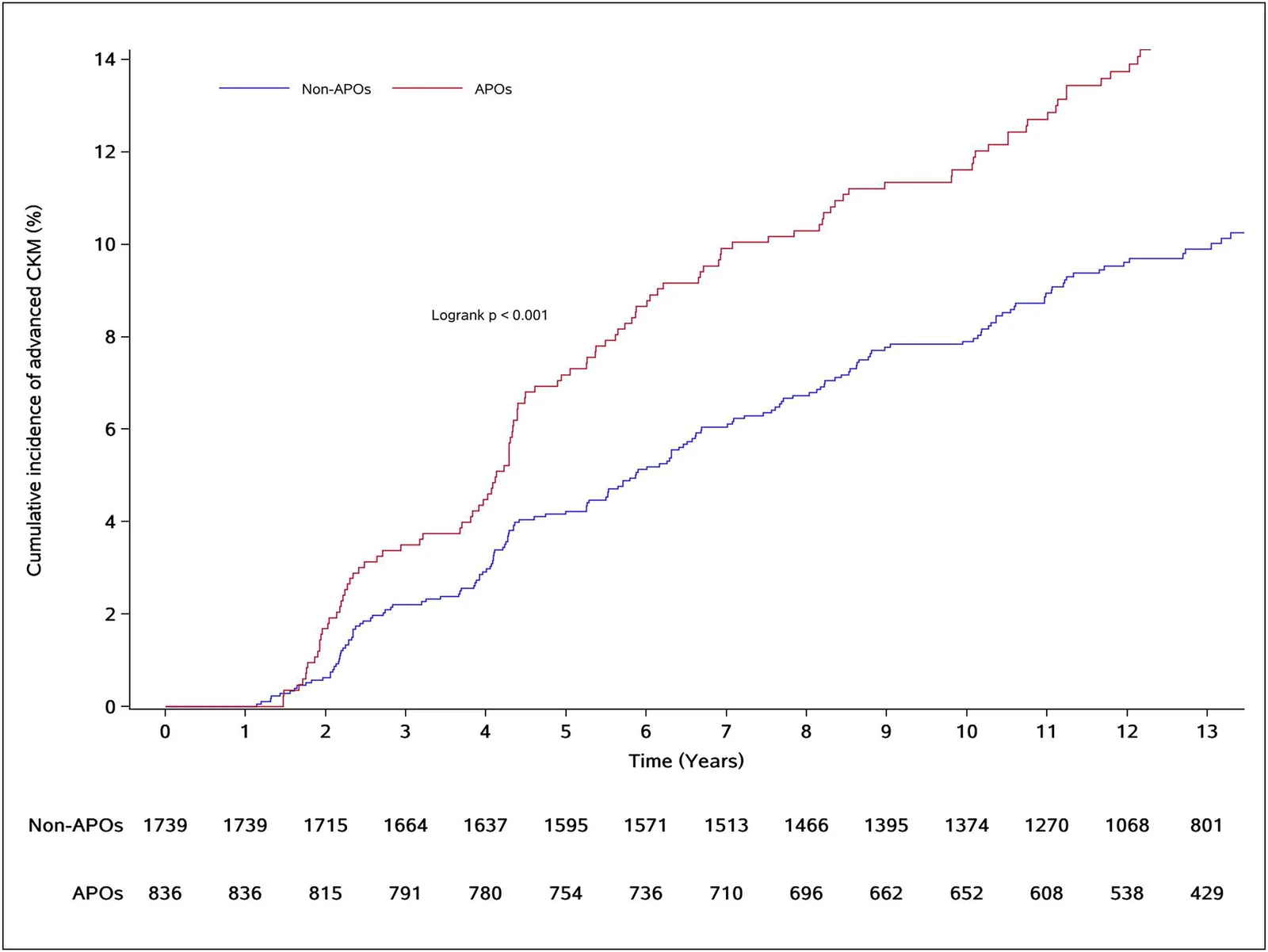
Cumulative incidence of advanced CKM in different groups.

After excluding participants with advanced CKM at baseline and those without any post-baseline health examination, 2,575 participants were included in the longitudinal analysis. During a median follow-up of 11.68 years, 302 participants (11.73%) developed advanced CKM. The incidence was higher in the APOs group than in the non-APOs group (13.39 vs. 8.42 per 1,000 person-years). Multivariable Cox regression analysis confirmed an increased risk of advanced CKM associated with APOs (HR, 1.27; 95% CI, 1.01–1.61) (Table 4). Kaplan–Meier analysis further showed a consistently higher cumulative incidence of advanced CKM in the APOs group throughout the follow-up period (Figure 2, log-rank *p* < 0.001).

**Table 4:** Cox regression analysis of the association between APOs and incident advanced CKM.

Groups	Incidence/total (number/number)	Incidence per 1,000 person-years	HR (95% CI)	
			Model 1	Model 2
Non-APOs	171/1,739	8.42	Reference	Reference
APOs	131/836	13.39	1.66 (1.32–2.09)	1.27 (1.01–1.61)

Model 1 was adjusted for age at delivery. Model 2 was further adjusted for age at baseline, low-density lipoprotein cholesterol, high-sensitivity C-reactive protein, uric acid, income level, salt intake, physical activity, and sleep duration based on Model 1.

APOs, adverse pregnancy outcomes; CKM, cardiovascular-kidney-metabolic.

### Sensitivity, stage-specific, and subgroup analyses

In sensitivity analyses, the association between a history of APOs and incident advanced CKM remained significant after excluding participants with less than 4 years of follow-up (HR, 1.33; 95% CI, 1.01–1.75; Table 5). Similar results were observed after excluding participants who were taking antihypertensive, glucose-lowering, or lipid-lowering medications at baseline (HR, 1.35; 95% CI, 1.06–1.73; Supplementary Table S2).

**Table 5:** Cox regression analysis of the association between APOs and incident advanced CKM after excluding participants with less than 4 years of follow-up.

Groups	Incidence/total (number/number)	Incidence per 1,000 person-years	HR (95% CI)
			Model
Non-APOs	121/1,637	6.04	Reference
APOs	94/780	9.74	1.33 (1.01–1.75)

The model was adjusted for age at delivery, age at baseline, low-density lipoprotein cholesterol, high-sensitivity C-reactive protein, uric acid, income level, salt intake, physical activity, and sleep duration.

APOs, adverse pregnancy outcomes; CKM, cardiovascular-kidney-metabolic.

We further examined CKM stages 3 and 4 as separate outcomes. Compared with women without a history of APOs, those with a history of APOs had an HR of 1.13 (95% CI, 0.87–1.46) for incident CKM Stage 3 and an HR of 1.60 (95% CI, 1.12–2.28) for incident CKM Stage 4 (Supplementary Table S3).

Subgroup analyses showed no significant effect modification by baseline age, BMI, education level, or income level on the association between a history of APOs and the risk of incident advanced CKM (Supplementary Figure S1).

## Discussion

By analyzing data from the Kailuan cohort, this study provides novel evidence that a history of APOs is associated with more severe CKM staging and an increased risk of disease progression in women. These findings extend the clinical significance of APOs beyond their established role as risk markers for single-system diseases, positioning them as early indicators of integrated multisystem decline. Consequently, pregnancy history should be recognized as a valuable sex-specific factor that can enhance early risk stratification and inform targeted preventive strategies against CKM in women.

Numerous studies have demonstrated that specific APOs are associated with the long-term risk of cardiovascular, kidney, and metabolic diseases, including CVD, CKD, and diabetes (27–29). However, evidence regarding the association between APOs and CKM, a multisystem syndrome, remains limited. In a cross-sectional study of 3,917 US women aged 20–49 years, Boyer et al. found that GDM and infertility were associated with a higher prevalence of CKM Stage 2 or higher, with adjusted prevalence ratios of 1.25 and 1.14, respectively (30). This study provided direct evidence linking female reproductive risk factors with CKM. However, it primarily used CKM Stage 2 or higher as the outcome and did not separately estimate the associations between reproductive risk factors and CKM stages 2, 3, and 4. In addition, the cross-sectional design precluded the assessment of temporality and incident CKM risk. Compared to the cross-sectional study by Boyer et al., the present study extends the evidence to Chinese women and examines the association between a composite measure of APOs and CKM. In the stage-specific cross-sectional analyses, a history of APOs was significantly associated with CKM Stage 1, CKM Stage 2, and advanced CKM, with ORs of 1.42, 1.65, and 2.93, respectively. The longitudinal analysis further showed that APOs were associated with an increased risk of incident advanced CKM (HR, 1.27; 95% CI, 1.01–1.61), providing longitudinal evidence for their long-term association. Although direct numerical comparisons are limited by differences in study populations, exposure and outcome definitions, study designs, and effect measures, the broadly consistent direction of associations across studies suggests that pregnancy-related and reproductive risk factors are associated with a greater CKM burden.

Notably, stage-specific analyses showed that APOs were significantly associated with an increased risk of incident CKM Stage 4 (HR, 1.60; 95% CI, 1.12–2.28), whereas the association with CKM Stage 3 was directionally consistent but did not reach statistical significance (HR, 1.13; 95% CI, 0.87–1.46). The crude incidence rate differences between women with and without a history of APOs were similar for CKM stages 3 and 4, at 3.05 and 3.04 cases per 1,000 person-years, respectively. However, among women without APOs, the incidence rate of CKM Stage 3 was higher than that of CKM Stage 4 (6.76 vs. 2.93 cases per 1,000 person-years); therefore, similar absolute rate differences may correspond to different relative effect estimates. In addition, CKM Stage 4 is characterized by established clinical CVD and therefore represents a more clearly defined disease phenotype, which may have made the association between APOs and CKM Stage 4 more readily detectable. Overall, these findings suggest that a history of APOs may be associated with an increased risk across different stages of CKM progression and may provide complementary information for long-term CKM risk assessment and early management in women.

From a biological perspective, pregnancy may act as a physiological stress test that unmasks preexisting cardiovascular and metabolic susceptibility while imposing additional vascular, renal, and metabolic stress, thereby contributing to subsequent CKM progression (2, 3). In HDP, particularly preeclampsia, abnormal placentation and uteroplacental hypoperfusion may induce angiogenic imbalance, oxidative stress, endothelial dysfunction, and glomerular injury, increasing the long-term risk of hypertension, kidney dysfunction, and CVD (31, 32). GDM reflects pregnancy-induced insulin resistance and inadequate pancreatic *β*-cell compensation, which may promote persistent dysglycemia, adiposity, dyslipidemia, and vascular injury (33). PTB and LBW may also reflect placental, vascular, or inflammatory dysfunction and may indicate underlying endothelial and microvascular vulnerability (12, 34). These shared pathways may converge through chronic inflammation, insulin resistance, endothelial injury, and renal microvascular dysfunction, thus providing a biologically plausible basis for CKM progression. At the molecular level, Talpur et al. reviewed epigenetic mechanisms linking pregnancy complications to subsequent cardiovascular risk in offspring, including alterations in DNA methylation, histone modifications, and non-coding RNA regulation (35). Related epigenetic alterations have also been observed in pregnancy complications, including altered DNA methylation in GDM and dysregulated non-coding RNAs in both preeclampsia and GDM (36–38). However, it remains unclear whether these epigenetic changes persist in maternal tissues and contribute to long-term CKM progression.

This study found that a history of APOs was associated with advanced CKM stages and an increased risk of incident advanced CKM, suggesting that obstetric history may provide additional information for the long-term assessment of cardiovascular, kidney, and metabolic health in women. In clinical practice, routine documentation of APOs may help identify women at an increased risk and facilitate a comprehensive evaluation of blood pressure, obesity-related measures, glucose and lipid metabolism, and kidney function, together with long-term monitoring and management of relevant risk factors.

This study used long-term follow-up data from a large community-based cohort, which reduced recall bias and supported the temporal interpretation of exposure–outcome associations. The CKM staging was based on internationally recognized criteria, enhancing comparability with other studies. We included a comprehensive set of covariates, applied multiple statistical models, and conducted sensitivity analyses to assess the consistency of the observed associations. However, the limitations of this study warrant further consideration. Although we adjusted for a range of baseline confounders, time-varying factors during follow-up such as lifestyle and medication use were not fully accounted for. Furthermore, subclinical CVD was defined based on the widely applied China-PAR risk model rather than imaging confirmation, which is common in large-scale epidemiological studies. Future studies incorporating repeated measures and imaging biomarkers could help further delineate risk profiles across APOs subtypes.

## Conclusion

In conclusion, this study showed that a history of APOs was associated with advanced CKM stages and an increased risk of incident advanced CKM. These findings suggest that obstetric history may provide complementary information for assessing women's long-term cardiovascular, kidney, and metabolic risks and support greater attention to APO history and long-term monitoring of related cardiovascular, kidney, and metabolic risk factors throughout the female life course.

## Data Availability

The raw data supporting the conclusions of this article will be made available by the authors, without undue reservation.
