# Supplementary material for: Association between adverse pregnancy outcomes and incidence of advanced cardiovascular-kidney-metabolic disease: a population-based cohort study
Source: Front Cardiovasc Med. 2026 Aug 21;13:1859791. doi: 10.3389/fcvm.2026.1859791 (PMC13541668; doi:10.3389/fcvm.2026.1859791)
Supplement: Supplementary file 1 [file Datasheet1.pdf]

Table S1 Baseline characteristics of the study population according to CKM stage

Characteristics	CKM 0 (N=432)	CKM 1 (N=414)	CKM 2 (N=1,794)	Advanced CKM (N=98)	*p*-value
Delivery age, years	26.5 ± 3.1	27.2 ± 3.8	27.5 ± 4.4	32.1 ± 5.6	<0.001
Baseline age, years	35.7 ± 6.0	39.1 ± 7.0	41.9 ± 7.4	51.6 ± 7.6	<0.001
WC, cm	69.7 ± 4.6	82.2 ± 9.1	82.8 ± 10.5	89.3 ± 9.5	<0.001
SBP, mmHg	104.2 ± 9.5	106.2 ± 9.6	121.2 ± 16.6	153.4 ± 27.0	<0.001
DBP, mmHg	68.9 ± 5.5	69.3 ± 6.0	79.7 ± 10.0	94.0 ± 15.3	<0.001
HDL-C, mmol/L	1.7 ± 0.2	1.6 ± 0.2	1.5 ± 0.4	1.6 ± 0.4	<0.001
LDL-C, mmol/L	1.9 ± 0.6	2.1 ± 0.7	2.2 ± 0.7	2.4 ± 0.7	<0.001
TC, mmol/L	4.6 ± 0.8	4.6 ± 0.8	4.8 ± 1.0	5.2 ± 1.2	<0.001
TG, mmol/L	0.7 (0.6, 0.9)	0.9 (0.7, 1.1)	1.1 (0.8, 1.7)	1.6 (1.0, 2.6)	<0.001
UA, μmol/L	227.3 ± 54.6	231.3 ± 56.6	232.3 ± 57.7	257.6 ± 64.5	<0.001
FBG, mmol/L	4.8 ± 0.4	5.1 ± 1.2	5.2 ± 1.5	7.2 ± 3.8	<0.001
BMI, kg/m^2	20.6 ± 1.4	24.5 ± 3.0	24.4 ± 3.6	27.3 ± 4.0	<0.001
hs-CRP, mg/L	0.4 (0.2, 1.2)	0.5 (0.2, 1.4)	0.5 (0.2, 1.2)	1.4 (0.6, 3.2)	<0.001
Physical activity, n (%)	22 (5.1%)	19 (4.6%)	70 (3.9%)	10 (10.2%)	0.025
High school or above, n (%)	288 (66.7%)	186 (44.9%)	449 (25.0%)	15 (15.3%)	<0.001
Income level ≥ 800 CNY/month, n (%)	125 (28.9%)	65 (15.7%)	160 (8.9%)	7 (7.1%)	<0.001
Salt intake, n (%)					<0.001
< 6 g/d	48 (11.1%)	21 (5.1%)	105 (5.9%)	9 (9.2%)	
6–10 g/d	351 (81.3%)	359 (86.7%)	1,597 (89.0%)	78 (79.6%)	

> 10 g/d	33 (7.6%)	34 (8.2%)	92 (5.1%)	11 (11.2%)	
Sleep duration, n (%)					<0.001
≤ 6 h/d	51 (11.8%)	38 (9.2%)	150 (8.4%)	17 (17.3%)	
6–8 h/d	363 (84.0%)	363 (87.7%)	1,621 (90.3%)	81 (82.7%)	
> 8 h/d	18 (4.2%)	13 (3.1%)	23 (1.3%)	0 (0.0%)	
Hypertension, n (%)	1 (0.2%)	6 (1.4%)	413 (23.0%)	79 (80.6%)	<0.001
Diabetes, n (%)	0 (0.0%)	7 (1.7%)	82 (4.6%)	36 (36.7%)	<0.001

Values are mean (standard deviation), median (interquartile range), or n (%) as appropriate.

BMI, body mass index; SBP, systolic blood pressure; DBP, diastolic blood pressure; FBG, fasting blood glucose; UA, uric acid; HDL-C, high-density lipoprotein cholesterol; LDL-C, low-density lipoprotein cholesterol; hs-CRP, high-sensitivity C-reactive protein; TC, total cholesterol; TG, triglycerides; WC, waist circumference.

CKM, cardiovascular-kidney-metabolic.

Table S2 Sensitivity analysis excluding participants using antihypertensive, glucose-lowering, or lipid-lowering medications at baseline

Groups	Incidence/Total (Number/Number)	Incidence per 1,000 person-years	*HR* (95% CI)
			Model
Non-APOs	145/1,664	7.39	Reference
APOs	120/793	12.89	1.35 (1.06–1.73)

The model was adjusted for age at delivery, age at baseline, low-density lipoprotein cholesterol, high-sensitivity C-reactive protein, uric acid, income level, salt intake, physical activity, and sleep duration.

APOs, adverse pregnancy outcomes.

Table S3 Associations of adverse pregnancy outcomes with incident CKM Stage 3 and CKM Stage 4

Endpoint	Incidence/Total (Number/Number)	Incidence per 1,000 person-years	*HR* (95% CI)
			Model
CKM Stage 3			
Non-APOs	138/1,739	6.76	Reference
APOs	97/836	9.81	1.13 (0.87–1.46)
CKM Stage 4			
Non-APOs	63/1,780	2.93	Reference
APOs	64/879	5.97	1.60 (1.12–2.28)

The model was adjusted for age at delivery, age at baseline, low-density lipoprotein cholesterol, high-sensitivity C-reactive protein, uric acid, income level, salt intake, physical activity, and sleep duration.

APOs, adverse pregnancy outcomes; CKM, cardiovascular-kidney-metabolic.

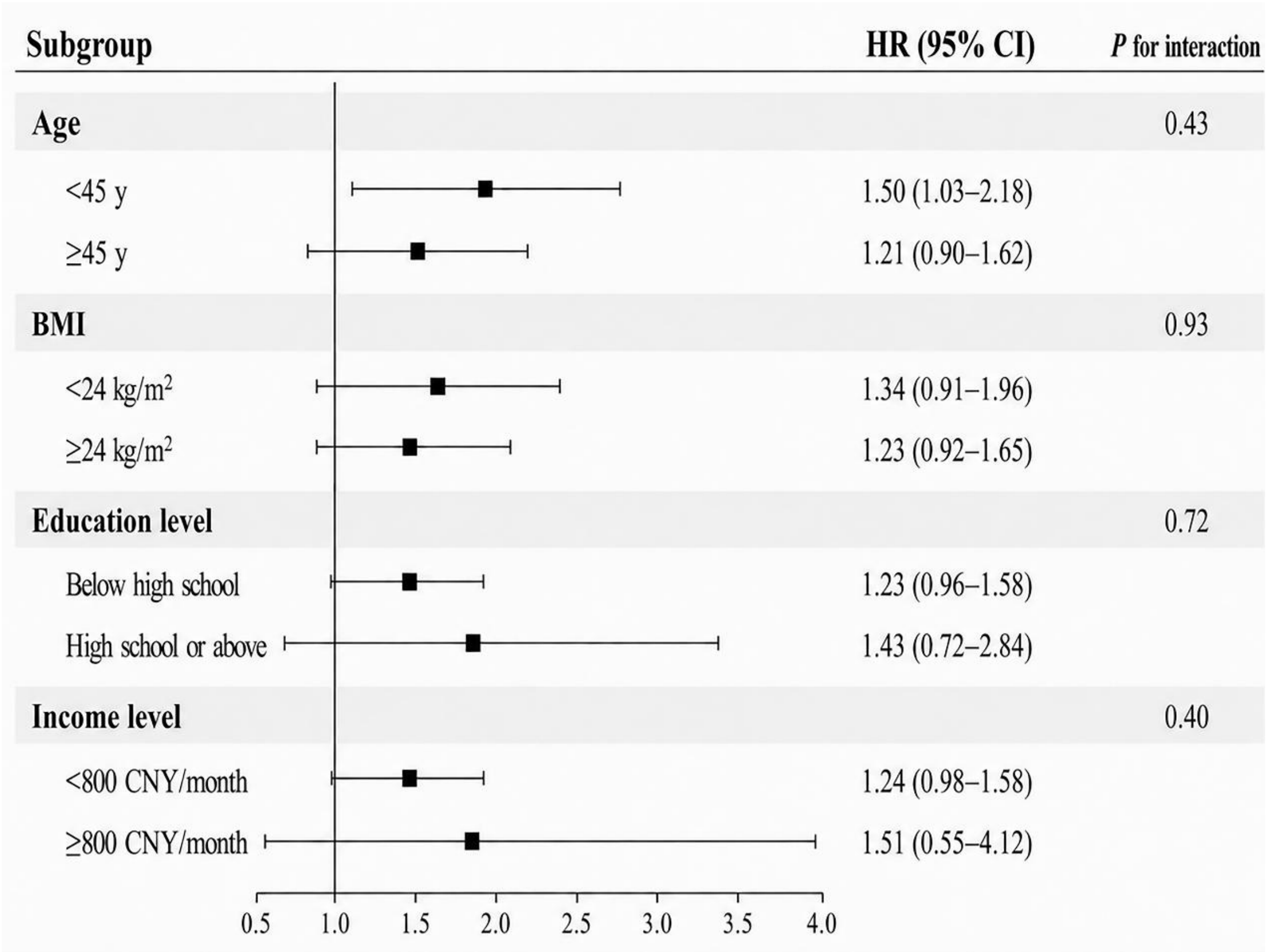

Figure S1 Subgroup analyses of adverse pregnancy outcomes and incident advanced CKM
